# Novel implementation research designs for scaling up global mental health care: overcoming translational challenges to address the world’s leading cause of disability

**DOI:** 10.1186/s13033-016-0049-7

**Published:** 2016-03-08

**Authors:** Susan M. Meffert, Thomas C. Neylan, David A. Chambers, Helen Verdeli

**Affiliations:** Department of Psychiatry, University of California, San Francisco, 401 Parnassus Avenue, San Francisco, CA 94127 USA; Department of Psychiatry, University of California, San Francisco, 4150 Clement Street, San Francisco, CA 94121 USA; National Cancer Institute, National Institutes of Health, BG 9609 MSC 9760, 9609 Medical Center Drive, Bethesda, MD 20892-9760 USA; Teachers College, Columbia University, 325 HMann 525 West 120th Street, New York, NY 10027 USA

**Keywords:** Mental health, Low and middle income countries (LMICs), Implementation science, Effectiveness-implementation hybrid, Interpersonal psychotherapy, HIV, Trauma, Depression, Treatment gap

## Abstract

Despite established knowledge that Low and Middle Income Countries (LMICs) bear the majority of the world’s burden of mental disorders, and more than a decade of efficacy research showing that the most common disorders, such as depression and anxiety, can be treated using readily available local personnel in LMICs to apply evidence-based treatments, there remains a massive mental health treatment gap, such that 75 % of those in LMICs never receive care. Here, we discuss the use of a new type of implementation science study design, the effectiveness-implementation hybrids, to speed the translation and scale up of mental health care in LMICs. We use our current study of Interpersonal Psychotherapy (IPT) delivered by local personnel for depression and trauma-related disorders among HIV+ women in Kenya as an example of effectiveness-implementation hybrid design for mental health services research in LMICs.

## Twenty years of rising mental disorder burden in LMICs

The 1996 global burden of disease (GBD) report by WHO and the World Bank was the first to put mental disorders on the list of highly disabling conditions. Twenty years later, the global disability attributed to mental disorders has increased by 45 % rendering them the leading cause of years lived with disability. While many low resource populations with unmet mental health care needs exist within high income countries (HICs), according to the GBD studies, the majority of the burden is carried by low and middle income countries (LMICs), where 75 % of those in need of mental health treatment never receive any care. The 2011 world economic forum projected that mental disorders would account for over half of the total economic burden from non-communicable diseases over the next two decades. The most common mental disorders in LMICs are depression and anxiety [including posttraumatic stress disorder (PTSD)] among adults [1], illnesses for which treatments with strong efficacy have been widely used in HICs for decades.

## Advances in treatment

### Non-specialists can treat common mental disorders in low resource settings

While the scarcity of mental health care providers was initially recognized as a contributor to high burden in LMICs, we now have a decade of studies showing that evidence-based treatments for depression and anxiety disorders, such as brief, structured psychotherapies, are feasible, acceptable and have strong effectiveness when delivered by local non-specialist personnel in LMICs [2]. However, with a few exceptions, most treatment studies in LMICs use traditional effectiveness designs, often foregoing valuable opportunities to deploy implementation science methodology—the goal of which is to identify practice and policy tools and strategies for successfully scaling up evidence-based interventions.

### Cost of inaction: the economics of mental health

Unlike diseases for which treatment costs contribute the majority of their economic burden, the costs associated with mental disorders are the costs of inaction. Depression, which is the most common mental disorder among adults, is notorious for its economic impact. Relative to other common diseases in working-age adults, depression has an earlier age of onset (often twenties) and higher chronicity. Depression impacts economic output through its association with work absenteeism (missing work) and decreased productivity (30 % decline with mild depression). *However, in HICs, depression and its associated economic losses are more than reversible with improved access to treatment. Studies have shown that investments in depression treatment have net return rates of over 300* *%* [3].

### An urgent need to scale

As global citizens, we have known for two decades that treatable mental disorders inflict disability on a massive number of people, the majority of whom reside in LMICs. We have known for one decade that evidence-based, effective, low cost treatments can be delivered by non-specialist personnel in low resource settings. We have strong data to suggest net positive returns on mental health care for depression, the most common mental disorder. *Yet, we have not succeeded in improving widespread access to evidence*-*based treatment for common mental disorders in LMICs.*

### Applying new implementation designs to global mental health: stimulating progress

Implementation science addresses the “know-do” gap in healthcare: the disparity between what scientific research identifies as the best evidence-based practices, and what is actually done in the community. Studies suggest that it takes an average of 17 years for 14 % of original research to be integrated into physician practice and that only 54 % of US adults receive care that meets indicators of high quality [4].

Implementation science improves the efficiency and impact of health care by informing the integration of evidence-based practices into clinical and community setting. Specifically, implementation researchers seek to disrupt use of the traditional “pipeline” model in which research progresses sequentially from basic science to treatment development and efficacy aiming to establish internal validity, followed occasionally by effectiveness and implementation studies to evaluate external validity. Implementation science focuses on the processes like financing, provider training and supervision, workflow and evidence-based practice demand through which efficacious interventions can be delivered within real-world settings. Implementation science questions can be integrated within efficacy and effectiveness trials to speed progression from treatment development to wide-spread use.

### Effectiveness-implementation hybrid research designs

Blending efficacy, effectiveness and implementation stages of research is a recent strategy to speed knowledge translation and produce evidence with greater relevance to practice and policy. Recently, Curran and colleagues defined effectiveness-implementation hybrid study designs [5]. Type I is recommended for situations in which the primary aim is to determine the effectiveness of an intervention when used with broad eligibility criteria, approximating “real world” use, with a secondary aim to better understand the context for implementation. Type II places equal weight on effectiveness and implementation aims, while Type III prioritizes investigation of the utility of an implementation intervention/strategy with secondary aims to evaluate the clinical outcomes associated with the implementation trial. Here, we provide an example of how effectiveness-implementation designs can be used to advance scale up of mental health care for common disorders in LMICs (Box 1).

### An effectiveness-implementation hybrid type I case example: HIV-positive women in Kenya

**Box 1:** The category fallacy

Medical anthropology has a major influence on the field of global mental health (GMH), with psychiatrist and medical anthropologist, Dr. Arthur Kleinman, conducting the first influential studies [6]. In some cases, anthropological research suggested that mental health diagnoses considered valid in one culture were not valid in populations that experienced and expressed emotions differently—the basis of the “category fallacy,” which led to debate about best practices in humanitarian aid and GMH treatment research. Partially reflecting the influence of medical anthropology, many treatment research studies are tightly indexed to the target population, often preceded by an ethnographically informed needs assessment—emphasizing assessment of the internal validity of diagnosis and treatment for the local/target community. While medical anthropology and ethnographic tools must remain cornerstones of an ethical approach to GMH treatment research, we suggest that *implementation science, particularly effectiveness*-*implementation study designs,* can build on this rich history, retaining a focus on internal validity while addressing today’s desperate need for broad scale up of mental health care for common disorders among diverse populations of adults in LMICs	

#### Setting

HIV infection in women is significantly associated with gender based violence (GBV) worldwide [7]. Survivors of GBV are at high risk of mental disorders, with 60–90 % developing posttraumatic stress disorder (PTSD) and/or depression [8]. In the setting of HIV, depression and PTSD not only cause suffering and disability, but correlate with deficits in adherence to antiretroviral therapy (ART). Our study site is the family aids care education and services (FACES) HIV care and clinical research clinic in the Nyanza region of Kenya, which has the highest national prevalence of HIV (19.3 %) and physical violence against women (57 % of women aged 15–49).

#### Needs assessment

In 2013, we completed a needs assessment study of HIV+ women affected by GBV (HIV+GBV+) women served by FACES. As reported elsewhere [9], more than half of participants described symptoms of depression, anxiety and traumatic stress among HIV+GBV+ women served by FACES. Participants identified problems with loss, transition and interpersonal conflict (Table 1). The coping skill identified by 82 % of study participants was social support from other HIV+ women. Women preferred to receive mental health treatment at the FACES clinic (versus separate location) and individual counseling was preferred over group treatment or medication.

**Table 1:** Optimizing IPT for HIV+GBV+ women in Kenya with MDD and PTSD

IPT stage	Adaptation
Summary of adaptations for meeting, inventory and formulation and termination phases	
Initial meetings (session 1–3)	Medical model of depression and PTSD in the setting of HIV, method and goals of IPT, interpersonal inventory including key components for study population: Disclosure of HIV status and its effects on relationships, GBV, housing and social support
Interpersonal formulation (sessions 4–5)	*Local examples*—
*Role conflict*	GBV, reproduction, condom use, HIV discordance in couple, inheritance
*Role transition*	HIV diagnosis, polygamy, single parenting, re-marriage, land disinheritance, separation from children
*Loss*	Family deaths secondary to HIV
Middle sessions (6–10)	Use local resources to advance social support around the identified problem area, including HIV women’s groups, women’s church groups and women’s chamas^a^
Concluding (sessions 11–12)	Review successes, relapse prevention strategies

^a^ *Chama* informal cooperative group that pools and loans funds to group members; *GBV* gender based violence; *HIV+* HIV-positive; *HIV+GBV+* HIV-positive women affected by gender based violence; *IPT* interpersonal psychotherapy; *PTSD* posttraumatic stress disorder

#### Treatment selection: interpersonal psychotherapy (IPT)

Given that many sources of emotional distress were related to interpersonal loss, transition or conflict, we hypothesized that IPT will be an effective treatment for depression and PTSD among HIV+GBV+ women served by the FACES clinic because it will improve communication and decision making around the identified problem area. Current coping skills, such as social support from other HIV+ women, suggest that IPT will be an acceptable treatment for this population, given that IPT mobilizes social support as a key strategy to enable effective management of the problem area. Our team and others have shown that IPT is feasible, acceptable and has strong efficacy in low resource settings, including delivery by non-specialists in sub-Saharan Africa.

**Box 2:** Global mental health (GMH) implementation science: next steps

*Study designs*. Encourage explicit use of implementation science in GMH treatment studies, leveraging new hybrid effectiveness-implementation designs at early stages of investigation Integrate within priority care systems Engage a reciprocal partnership with local and national policy makers and opinion leaders as well as health and mental health practitioners and researchers in the early stages of the treatment study, focused on scaling up mental health care to address local needs Continue and extend the GMH history of context-dependent adaptations of interventions to improve fit with population needs and service setting Develop criteria for selection of study personnel for sustained, collaborative implementation Use explicit strategies to develop local, sustainable methods of supervising non-specialist providers *Study outcomes*. Emphasize policy-relevant outcomes for GMH: Evaluate treatment effect on health co-morbidities (e.g., HIV viral load, neurocognitive deficits and other communicable and non-communicable diseases) Conduct cost analyses (e.g., cost-benefit and changes in economic productivity)	

#### Adaptation, training and manual

Evidence-based treatments are not always designed to be implemented in settings where they are most needed, requiring adaptation for the target population. Given the influence of culture on emotions, adaptation is often a key aspect of global mental health (GMH) treatment research (Box 2). In our adaptation of IPT, we focused on content and process, using data from our needs assessment study with further adaptations during on-site training of prospective IPT therapists (local non-specialists) and pilot IPT cases (Table 1).

#### Study design

We are now using an effectiveness-implementation hybrid type I study design to deliver IPT integrated within the HIV care platform, in order to move efficiently toward implementation and scale up of mental health care, while monitoring individual clinical outcomes [5]. The hybrid type I design prioritizes evaluation of the effectiveness of the intervention (Table 2) with participant level randomization, but allows for data collection on implementation parameters (Table 3).

**Table 2:** A type I effectiveness-implementation hybrid trial design for global mental health: effectiveness

Study component	Description
Treatment effectiveness: randomized controlled trial (RCT) within a routine clinical setting with minimal restrictions	
Target population	HIV+ women affected by GBV with MDD and PTSD, enrolled in HIV care at the UCSF-KEMRI FACES clinic supported by PEPFAR, which treats >140,000 HIV+ individuals in the Nyanza region of Kenya
Recruitment	Study information provided in waiting area for self-referral, HIV clinic providers alerted to the study and eligibility criteria
Eligibility	HIV-infected women over age 18, enrolled in HIV care at FACES, PTSD secondary to GBV and MDD, absence of cognitive dysfunction, severe mood/thought disorders and substance abuse requiring a higher level/alternate care (qualitative needs assessment suggested that these criteria would identify a high proportion of HIV+GBV+ women in need of mental health care at FACES)
Intervention	Participants will be randomized to receive: [1] 12 sessions of weekly IPT delivered at the FACES clinic plus FACES psychosocial treatment as usual (TAU) or [2] FACES TAU; TAU group is offered IPT at week 12
Concurrent treatment	Any mental health counseling/psychotherapy, psychotropic medication, ARV adherence counseling, couples therapy, other study participation and/or other psychosocial intervention at the FACES clinic or outside is allowed and noted
Retention	For missed sessions or evaluations, participants are called up to four times and emergency contact is alerted
RCT outcomes	*Primary:* diagnosis of MDD/PTSD; *Secondary:* continuous measures of depression and PTSD symptoms, interpersonal functioning, anger, self-efficacy, substance use, quality of life, disability, HIV viral load, self-reported ARV adherence and neurocognitive functioning. Primary and secondary outcomes assessed at baseline and repeated at weeks 12, 24, 36
IPT adaptation and therapist training	Adaptations to IPT content and process to optimize fit while maintaining fidelity to IPT protocol, drawing on prior experience with IPT adaptation. Additional IPT adaptations were made based on feedback from therapist non-specialist trainees during 2 week formal IPT training and 12 week pilot cases
Adherence to protocol	Evaluated after each session by an IPT study supervisor, using a session-specific IPT adherence monitoring, consisting of 9–10 items scored on a 10 point likert scale, including a reverse coded item. All sessions are audio-recorded and a random 20 % of sessions are evaluated by an independent rater
Sample size	220
Data analysis	Main analysis is comparison of change from baseline to post-treatment (12 weeks) between IPT+TAU and TAU. Maintenance of gains assessed by testing for significant change from 12 week to 24 and 36 week follow up assessments. Sub-group (sensitivity) analyses will be used to identify sub-groups for whom IPT+TAU is more or less effective

*ART* anti-retroviral therapy; *FACES* family AIDS, care, *GBV* education and services; gender based violence; *HIV+* HIV-positive; *IPT* interpersonal psychotherapy; *KEMRI* Kenya medical research institute; *MDD* major depressive disorder; *PEPFAR* president’s emergency plan for AIDS relief; *PTSD* posttraumatic stress disorder; *TAU* treatment as usual; *UCSF* University of California, San Francisco

**Table 3:** A Type I effectiveness-implementation hybrid trial design for global mental health: implementation

Implementation factor	Goal	Strategy
Treatment implementation		
Study location	Deliver mental health care in patients’ preferred manner using a system that can be taken to scale	Integrate mental health treatment within the HIV clinic, with clear delineation of the treatment pathway, including case identification, treatment, discharge and referral decision rules
Study personnel	Promote knowledge and integration of mental health care within the HIV clinic	Employ clinic staff as study personnel when possible
Clinic staff involvement	Engage clinic staff in a dialogue on the need for and benefits of mental health care within the HIV clinic and develop a common understanding of potential facilitators and barriers to treatment	Key clinic staff serve as study advisors and attend weekly meeting—e.g., IPT peer supervision is attended by leaders of the clinic’s ARV adherence team^a^
Study treatment personnel	Evaluate the success of implementing mental health treatment delivered by local non-specialists	Train and employ local, non-specialists to provide low cost, mental health care
Supervision	Build sustainable, local IPT supervision	IPT study therapists are supervised by IPT experts and by a weekly peer group of study therapists. During the study, supervision responsibility is transferred from experts to the local peer group
Sampling frame	Optimize applicability of study	Broad eligibility
Non mental health outcomes	Identify key correlates of mental health treatment:	
	HIV health	HIV health outcomes: viral load, ART adherence
	Cognitive function	Neurocognitive testing
	Economic gains	Cost-benefit analyses of mental health care for HIV+ women, including changes in formal and informal income
	Psychosocial	Quality of life, functionality, re-victimization
	Identify participant, therapist and clinic experience with delivering mental health treatment, including burden to clinic staff and suggestions for improvement	Qualitative interviews throughout and at the conclusion of the study, with integration of feedback to optimize treatment implementation parameters
Policy maker involvement	Collaborate with policy makers to create a scalable mental health treatment for HIV+ women in Kenya	Meet with local policy makers and invite them to the study, identify their data needs for scaling up mental health care, work to meet these needs
Refinements for scale up	Refine treatment, delivery and stakeholder involvement to optimize the intervention for national scale up	Formative evaluation of using qualitative exit interviews with study participants, therapists, clinic staff, policy makers and other stakeholders

*ART* anti-retroviral therapy, *IPT* interpersonal psychotherapy

^a^The clinic’s ART adherence team leader and other members were identified during the needs assessment and training period as local experts in psychosocial needs and emotional communication with the clinic’s patients

## Summary and next steps

Despite evidence that common and treatable mental disorders are the leading global cause of disability and repeated calls for broad-scale implementation of mental health services in LMICs, GMH implementation research remains nascent. New effectiveness-implementation study designs allow for monitoring of clinical outcomes, while advancing research to support mental health care scale up. GMH now has: [1] twenty years of data on the burden (including the economic burden) of mental disorders in LMICs; [2] a rapidly growing body of acceptable, feasible, culturally relevant and effective treatments for depression and anxiety delivered by local non-specialist personnel; [3] access to emerging effectiveness-implementation study designs that can be incorporated into early stage treatment research to guide eventual scaling-up. Now, more than ever, global health funders and global mental health researchers have the incentives and tools to partner with government, academic and opinion leaders and use implementation science to scale up mental health care services for common mental disorders in LMICs.
